# Endogenous oxytocin-linked heart-rate synchrony and social connection during live sports spectatorship

**DOI:** 10.1038/s41398-026-04265-2

**Published:** 2026-07-08

**Authors:** Takashi Matsui, Takafumi Yamaguchi, Daisuke Funabashi, Shion Takahashi, Hiroki Matsuoka, Ren Takamizawa, Yukina Tachibana, Shohei Dobashi, Kiyonobu Kigoshi, Shinzo Yamada, Hideki Takagi

**Affiliations:** 1 Institute of Health and Sport Sciences, University of Tsukuba, 1-1-1 Tennodai, Tsukuba, Ibaraki 305-8574 Japan; 2 R&D Center for Sport Innovation, University of Tsukuba, 1-1-1 Tennodai, Tsukuba, Ibaraki 305-8574 Japan; 3 Bureau of Physical Education and Sport, University of Tsukuba, 1-1-1 Tennodai, Tsukuba, Ibaraki 305-8574 Japan

**Keywords:** Human behaviour, Physiology

## Abstract

Oxytocin is a putative pharmacological target for social dysfunction. However, clinical trials of intranasal oxytocin have produced modest and inconsistent effects, motivating the search for naturalistic, non-pharmacological ways to recruit endogenous oxytocin. Here we show that a single session of live collegiate sports spectating is associated with endogenous oxytocin and interpersonal heart-rate (HR) synchrony, both of which were linked to psychological indices of social bonding among casual fans. Sixty adult spectators, including 21 female participants, provided repeated saliva samples for oxytocin and cortisol, continuous HR recordings, and ratings of enjoyment and unity before, during, and after a basketball game. Oxytocin dynamics were baseline dependent: individuals with low pre-game oxytocin showed clear increases, whereas those with high pre-game oxytocin maintained elevated concentrations; meanwhile, cortisol decreased and the oxytocin-to-cortisol ratio increased. HR synchrony also increased during and after the game. Notably, endogenous oxytocin levels showed a slight but positive association with the degree of HR synchrony, linking endocrine dynamics to interpersonal physiological coupling. In turn, higher oxytocin levels and stronger synchrony were associated with greater enjoyment, stronger unity, and intention to revisit, with perceived unity partially mediating links between physiological responses and experiential outcomes. These findings suggest that coordinated salivary oxytocin dynamics and physiological synchrony are candidate socio-physiological processes associated with social connection during live sports spectating. Live sports events may therefore serve as simple, scalable, non-pharmacological contexts in which oxytocin-related social experiences naturally arise in everyday life.

## Introduction

Oxytocin is a hypothalamic neuropeptide with potent effects on social cognition and stress regulation, and has attracted sustained interest as a pharmacological target for social dysfunction across neuropsychiatric conditions, including autism spectrum disorder and schizophrenia [1–3]. Intranasal oxytocin can acutely modulate social attention, emotion recognition, and limbic–striatal activity, motivating its clinical evaluation as an adjunctive approach to social impairments [4–6]. However, large-scale and longer-term trials have yielded small or inconsistent benefits on core social outcomes, and key uncertainties remain regarding dosing schedules, individual response profiles, and long-term safety [7–9]. In addition, peripheral administration faces practical constraints, including limited central penetration, short half-life, and potential off-target peripheral effects [10, 11]. Together, these limitations highlight a translational need for scalable, non-pharmacological strategies that can engage endogenous oxytocin-linked systems in everyday life, without relying on exogenous administration [12, 13].

Endogenous oxytocin is released by a range of affiliative cues and embodied social behaviors. Low-intensity, non-noxious social touch (e.g., gentle stroking or massage) has been linked to stress-buffering effects and increases in peripheral oxytocin, alongside engagement of cortical regions implicated in reward and social cognition [14–16]. Structured practices that combine physical exertion with dyadic contact, such as martial arts, can also increase salivary oxytocin, with responsivity varying by interaction form and proximity [17, 18]. Beyond touch, social vocalizations can facilitate oxytocin release and accelerate cortisol recovery after stress [19, 20], and naturally occurring affiliative contexts such as early-stage romantic attachment show elevated oxytocin that covaries with interactive reciprocity and dyadic synchrony [21]. At the same time, a recent meta-analysis indicates that endogenous oxytocin shows only a modest overall association with social interaction, with substantial heterogeneity across individuals, contexts, and measurement approaches [22, 23]. One plausible contributor to this variability is pre-existing neuroendocrine state. Baseline oxytocin may reflect tonic regulatory set-points that shape subsequent hormonal responsivity to salient social experiences [24]. Consistent with this perspective, prior studies have compared individuals with relatively lower versus higher baseline oxytocin levels when examining differential responses in social-cognitive and stress-related contexts [25, 26]. Under this framework, individuals exposed to the same collective event may show different oxytocin dynamics—such as increases, maintenance, or attenuation—while still converging on broadly shared emotional and interpersonal outcomes. Moreover, oxytocin is closely linked to HPA-axis regulation, indicating that broader endocrine balance may complement oxytocin-specific measures when characterizing responses to social experiences [27, 28]. This heterogeneity underscores the importance of identifying ecologically valid, broadly accessible social behaviors that can robustly recruit endogenous oxytocin under real-world conditions [22].

Large-scale collective gatherings—religious rituals, concerts, and sporting events—can generate intense shared emotional experiences and feelings of togetherness, and physiological synchrony in crowds has been proposed as one mechanism linking collective experience to social bonding [29, 30]. Field and laboratory studies show that heart-rate (HR) synchrony increases in dyads and crowds, and that greater synchrony predicts stronger connectedness and cooperative orientations [31, 32]. Intranasal oxytocin has been reported to enhance physiological linkage and inter-brain synchrony during dyadic and small-group interaction [33, 34], but it remains unclear whether naturally fluctuating endogenous oxytocin covaries with interpersonal HR synchrony in ecologically valid collective settings. In the domain of sport fandom, the Team Identification–Social Psychological Health Model posits that identification and spectatorship cultivate social connections that support well-being [35], and observational and longitudinal evidence links live sport spectatorship and loyalty with greater life satisfaction, perceived emotional support, and reduced loneliness, particularly among middle-aged and older adults [36–40]. Yet from a neuroendocrine perspective, oxytocin in spectators remains largely unexplored: research in sport settings has focused mainly on cortisol and testosterone in athletes and (to a lesser extent) fans [41, 42], leaving open how endocrine dynamics relate to crowd-level physiological coupling and momentary changes in perceived social connection during real-world events. It remains unclear whether endogenous oxytocin covaries with interpersonal HR synchrony and whether their coupling is linked to social-connection-relevant outcomes among casual spectators.

Here we addressed this gap by testing whether a single session of live sports spectatorship recruits endogenous oxytocin dynamics and interpersonal HR synchrony in association with social bonding and experiential outcomes in everyday life. We hypothesized that (i) spectatorship is associated with baseline-dependent oxytocin dynamics alongside a shift toward an oxytocin-dominant endocrine balance (indexed by reduced cortisol and an increased oxytocin-to-cortisol ratio), (ii) spectatorship increases HR synchrony among co-present spectators, and (iii) oxytocin levels covary with the degree of synchrony, with higher oxytocin and stronger synchrony being associated with greater enjoyment and perceived unity and, in turn, with downstream experiential and motivational outcomes (flow and intention to revisit). To test these hypotheses in an ecologically valid setting, we conducted a prospective observational field study of casual spectators attending collegiate basketball games, integrating repeated salivary oxytocin/cortisol sampling with continuous HR recording and repeated assessments of enjoyment and unity before, during, and after the event.

## Materials and methods

### Participants

**Fig. 1:**
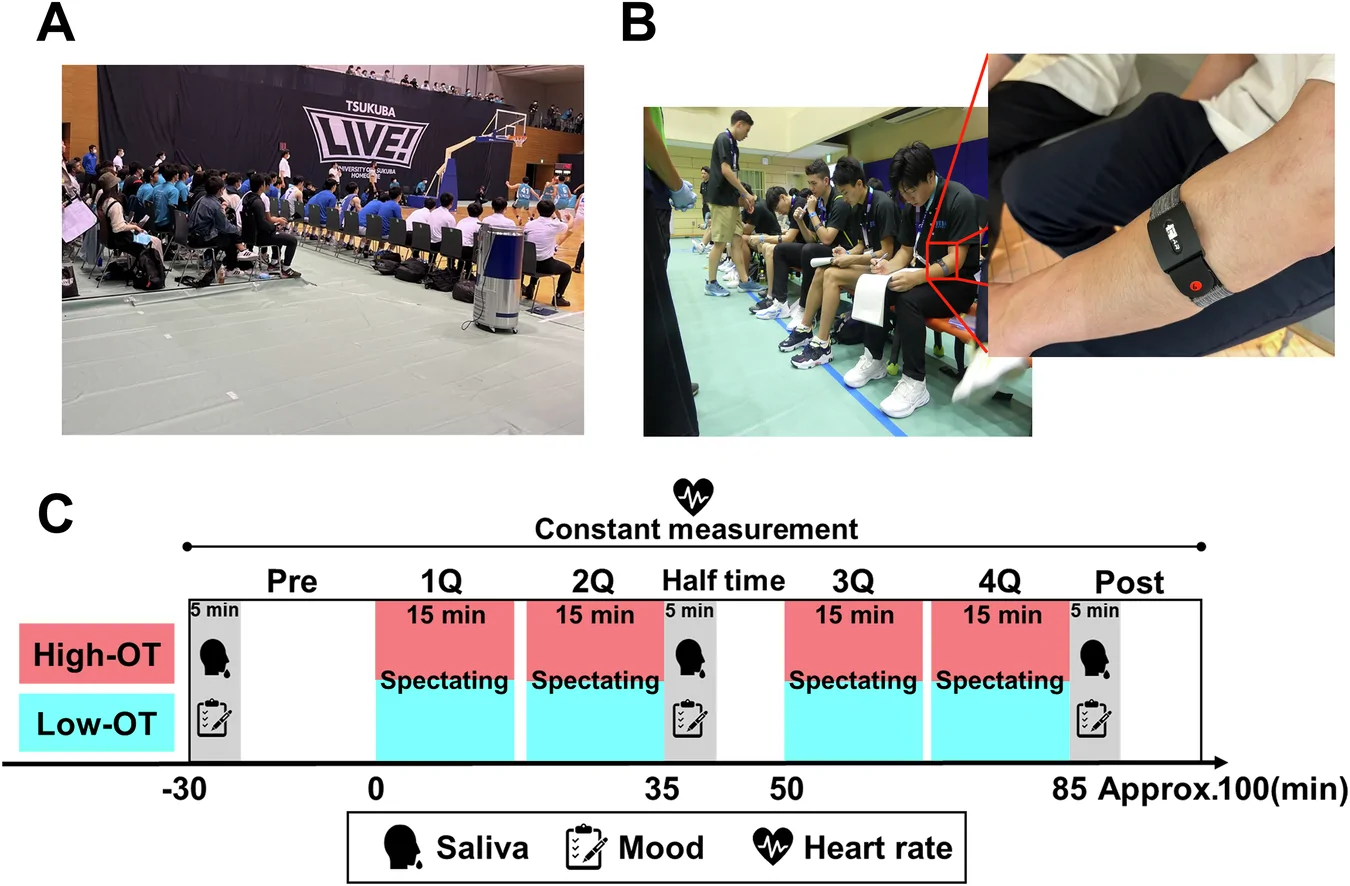
The experimental protocol. **A** A typical photo of the spectating state. **B** A typical photo of saliva sampling, questionnaire completion, and the HR measurement device. **C** Overview of the experimental protocol. The participants watch basketball games for approximately 100 min. Saliva samples and mood questionnaires were administered before the game, at halftime, and after the game. Each measurement took approximately 5 min to complete. The HR was measured during spectating.

We conducted a prospective observational field study with spectators attending home basketball games at the University of Tsukuba. A total of 62 adults were recruited from attendees of “TSUKUBA LIVE!”, the university’s home event series (Fig. 1A). Unlike professional sports events, these collegiate games attracted modest crowds primarily composed of students and local residents, reflecting a predominantly casual rather than highly devoted fan base (according to a pre-match survey, there were no fans who had been passionately supporting the home team for some time). Each participant attended one game, which resulted in either a win or a loss for the home team.

**Fig. 2:**
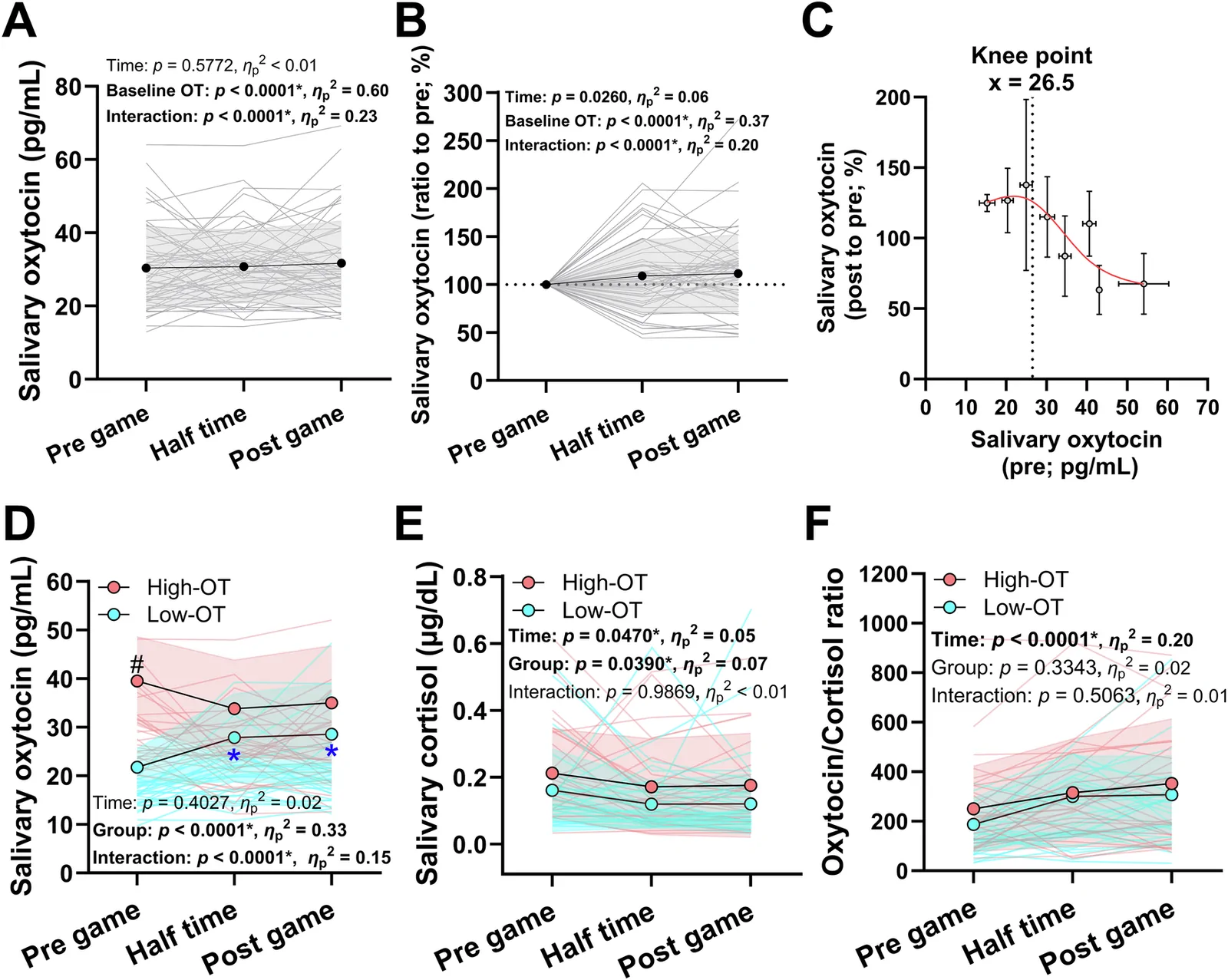
Endogenous oxytocin dynamics shift in a baseline-dependent manner during live sports spectating. **A** Absolute salivary oxytocin concentration (n = 60). **B** Individual oxytocin ratios relative to pre-game levels for all participants (n = 60). **C** Restricted cubic spline model illustrating the non-linear relationship between baseline oxytocin levels and their subsequent rate of change. **D** Temporal changes in salivary oxytocin. **E** Salivary cortisol. **F** Oxytocin-to-cortisol ratio. The High-OT group is shown in red (n = 33) and Low-OT group is shown in blue (n = 27). Data points represent group means, with shaded bands representing the standard deviation (SD) and thin lines representing individual trajectories. Results of two-way mixed-model ANOVA (factors: Time, Group, and Interaction) are indicated in the lower-left corner of each panel. Post-hoc comparisons were performed using Bonferroni’s multiple comparison test: *p < 0.05 vs. pre-game for both groups; # p < 0.05 vs. Low-OT group at the same point. The High-OT and Low-OT grouping is shown for descriptive visualization only; primary analyses treated baseline oxytocin as a continuous predictor.

Two participants were excluded from all analyses due to insufficient pre-game saliva samples, leaving 60 participants in the final analytic sample. To identify a physiologically relevant threshold for grouping instead of median splitting, which leads to statistical information loss [43], we performed a restricted cubic spline (RCS) analysis on the relationship between baseline oxytocin and post-game change rates [44] (detailed in the Statistical Analysis section and Fig. 2C). Based on the identified mathematical inflection point (Knee point; 26.5 pg/mL; Supplementary Table 1 & Supplementary Fig. 1), participants were stratified into a high baseline oxytocin group (High-OT; n = 33; 22.1 ± 8.3 years; 10 females) and a low baseline oxytocin group (Low-OT; n = 27; 23.1 ± 9.3 years; 11 females). Baseline oxytocin levels were higher in the High-OT group than in the Low-OT group by design, whereas there were no significant group differences in sex, age, body composition, resting HR, or habitual physical activity, indicating that the grouping primarily reflected pre-game oxytocin concentration rather than broader demographic or physiological differences (Supplementary Table 1).

A priori power analysis was conducted using G*Power 3.1 for a two-way mixed design ANOVA. Based on a partial η² of 0.036 (f = 0.19) derived from a previous study on baseline-dependent oxytocin reactivity [45], this analysis indicated that a total sample size of 60 participants would provide 90% power to detect a significant interaction effect with an α level of 0.05, assuming a correlation of 0.5 among repeated measures.

Inclusion criteria were as follows: age 18 years or older; ability to understand the study procedures and provide informed consent; no current medication for neuropsychiatric, cardiovascular, or metabolic diseases; and ability to attend the stadium for the duration of the measurements. The study was approved by the Research Ethics Committee of the Institute of Health and Sport Sciences, University of Tsukuba, and was conducted in accordance with the Declaration of Helsinki. All participants provided written informed consent prior to participation. The study is reported in accordance with the STROBE guidelines for observational studies.

### Study design and procedure

This prospective observational field study was conducted during two home basketball games at the University of Tsukuba (Fig. 1C). Both games took place in the late afternoon to evening. This timing was specifically selected to reflect the naturalistic scheduling of real-world sporting events, thereby maximizing the ecological validity of the spectatorship experience. The study was designed to capture coupled endocrine and cardiovascular responses to live sports spectating by combining repeated saliva sampling with continuous HR recording and self-report measures of mood and social connection.

When inviting participants, an outline of the study and a URL link to an online pre-survey (see Section 2.3) were sent via e-mail to potential spectators of the home event series “TSUKUBA LIVE!”. Participants were instructed to refrain from consuming alcohol from the day before the game until the end of the experiment, to avoid caffeine intake and strenuous physical exercise on the day of the game, and to abstain from eating for at least 2 h before arriving at the stadium.

On the day of measurement, participants arrived at the indoor arena approximately 60 min before tipoff. Upon arrival, they received a detailed explanation of the procedures again and provided written informed consent. Participants were then fitted with an optical HR sensor (Polar Verity Sense; Polar, Finland) on the upper arm and completed baseline saliva sampling (2 mL) for hormone analysis as well as baseline psychological questionnaires in the concourse area (Fig. 1B). After these baseline assessments, participants moved to their assigned seats and watched the game under naturalistic conditions. Additional saliva samples and questionnaires were collected at halftime and immediately after the game, yielding three repeated measurement points: pre-game (T1), halftime (T2), and post-game (T3). Participants were not permitted to smoke during the game. Information regarding hormonal status (e.g., menstrual cycle phase and hormonal contraception use) was not systematically controlled, to preserve ecological validity in the naturalistic spectating environment.

### Demographic and physical activity assessment

Before the game, participants completed an online pre-survey delivered via Google Forms to collect demographic characteristics (for example, age and sex) and habitual physical activity. Habitual physical activity was assessed using the Japanese version of the International Physical Activity Questionnaire (IPAQ), which estimates average daily time spent in vigorous-intensity, moderate-intensity, and walking activities, as well as sedentary behavior.

Some participants did not complete all demographic or physical activity items, resulting in missing data for certain questionnaire variables. These missing values were not imputed. Analyses involving those specific variables were conducted using available-case data, whereas participants’ physiological (HR, salivary hormones) and psychological (questionnaire) measures were retained for all other analyses when data quality met the inclusion criteria (age, diseases, availability of baseline oxytocin levels). Missing values were observed in questionnaire variables ranging from 1 (1.7%) to 6 (10%), and 1 (1.7%) sample had missing heart rate data. Participants with missing data did not show clear group differences in available demographic or physiological characteristics.

### Saliva collection and oxytocin measurement

Salivary oxytocin and cortisol were measured as neuroendocrine markers indexing social bonding and stress-related responses during spectating. At each time point (pre-game, halftime, post-game), participants provided approximately 2 mL of whole saliva by passively drooling through a straw directly into polypropylene collection tubes. All saliva samples, including those collected at halftime, were immediately stored in an ice box filled with dry ice after collection and transferred to a − 80 °C deep freezer within 3 h of collection, where they were stored until processing. To precipitate mucins and other debris, frozen samples were thawed and centrifuged at 1500 × g for 20 min; the clear supernatant was then aliquoted and re-frozen at −80 °C until assay.

For oxytocin quantification, 1000 μL of each saliva sample was concentrated by centrifugal evaporation and reconstituted according to the manufacturer’s instructions before assay with a commercial competitive enzyme-linked immunosorbent assay (ELISA) kit (Oxytocin ELISA Kit, Enzo Life Sciences, USA). Samples were reconstituted to achieve a fourfold concentration relative to the original saliva sample, and oxytocin concentrations obtained from the assay were back-calculated to the original saliva concentration by dividing by the concentration factor. According to the manufacturer, the oxytocin assay had a standard curve range of 15.6–1000 pg/mL, a detection limit of 15.0 pg/mL, and inter-assay coefficients of variation of 20.9%, 16.5%, and 11.8% at low, medium, and high concentration ranges, respectively. Salivary cortisol concentration was measured using a high-sensitivity ELISA kit (Salivary Cortisol ELISA Kit, Salimetrics, USA). According to the manufacturer, the analytical sensitivity of the cortisol assay was 0.007 μg/dL, the functional sensitivity was 0.018 μg/dL, the lowest standard was 0.012 μg/dL, and inter-assay coefficients of variation ranged from 3% to 11% depending on concentration level.

All samples were assayed in duplicate on 96-well plates, and absorbance was read using a multimode microplate reader (Varioskan LUX, Thermo Fisher Scientific, USA). Hormone concentrations were calculated from standard curves generated for each plate. To minimize potential plate effects, samples from the same participant (pre-game, halftime, and post-game) were assayed on the same plate, and samples were distributed across multiple plates so that the experimental groups were not unevenly represented on any plate. Across all assays, the coefficient of variation for duplicate determinations was below 5%, indicating acceptable analytic reliability. All samples yielded concentrations above the analytical sensitivity of the assays.

### HR measurements

To evaluate the physiological impact of live sports spectating, we assessed HR as a cardiovascular activity index. HR was monitored continuously using a standalone optical HR sensor worn on the upper arm (Polar Verity Sense; Polar, Finland) [46], as described in Section 2.2. Participants wore the sensor from approximately 30 min before tipoff until 15 min after the end of the game. HR data were sampled at 1 Hz throughout this period. The device-processed HR values exported from the sensor were used for subsequent analyses. Before synchrony analysis, HR recordings were inspected for missing values, signal dropouts, and physiologically implausible outliers. The spectating period was then segmented into “in-game” intervals (when play was in progress and players were on the court) and “out-of-game” intervals (inter-quarter breaks, halftime, and the 15-minute post-game period).

### Sensitivity analysis of HR synchrony analysis

HR synchrony between spectators was quantified using cross-correlation function (CCF) analysis of the HR time series [47]. In this method, the CCF is calculated for each pair of participants across a predefined temporal lag window to identify the maximum correlation between their heart rate time series and the lag at which this maximum occurs. Since the choice of lag window may influence estimates of synchrony, a sensitivity analysis was conducted prior to the main heart rate synchrony analysis to determine appropriate lag window conditions. Specifically, heart rate synchrony was calculated under multiple lag window conditions, and the resulting synchrony indices were correlated with behavioral measures. To facilitate comparison between lag window conditions, correlation coefficients were transformed using Fisher’s z-transformation. The lag window conditions adopted for the primary heart rate synchrony analysis were determined by comprehensively considering the results of this sensitivity analysis and the temporal structure specific to basketball possessions [48].

### HR synchrony analysis

For each participant pair, CCFs were computed within a temporal lag window of ±30 s to identify the maximum correlation between their HR time series. The temporal lag at which the maximum CCF occurred was extracted, and the absolute value of this lag was taken as an index of the degree of temporal alignment irrespective of direction, with smaller values indicating tighter alignment. In this study, we assumed that synchronization stems primarily from shared external stimuli rather than stable, directional interpersonal influences. Therefore, we did not model leader-follower relationships between individuals, but instead focused on the magnitude of temporal lags, regardless of their direction.

Because participants were seated in two separate blocks on opposite sides of the same courtside, their visual perspectives of the game could differ and potentially influence synchrony. To account for this, synchrony scores were calculated within each seating block rather than across all participants. For each participant, an individual synchrony score was obtained by averaging the dyadic CCF values across all pairings with other participants in the same seating block.

Baseline HR synchrony was defined as the 5-minute interval during the pre-game period that showed the lowest within-block CCF values, and synchrony indices during spectating were expressed relative to this baseline where appropriate. In-game and out-of-game synchrony measures were derived from the segmented HR data described in Section 2.5, enabling us to examine whether physiological synchrony differed between active play and non-play intervals and how these dynamics related to neuroendocrine and psychological measures.

To examine whether the observed physiological alignment was context-specific rather than attributable only to nonspecific stimulus-driven covariance, we conducted surrogate control analyses (pseudo-synchrony). We first calculated HR synchrony between randomly paired participants who attended different games (inter-game synchrony) and compared it with synchrony among participants who attended the same game (intra-game synchrony). We then compared synchrony among participants seated in the same block (intra-block synchrony) with synchrony among participants seated in different blocks (inter-block synchrony) to evaluate the influence of shared local visual and auditory context.

### Psychological questionnaires

Subjective experiences during spectating were assessed using four self-report measures: enjoyment, flow state, intention to revisit, and perceived social unity.

#### Enjoyment

Momentary enjoyment while watching the game was assessed using a 100-mm visual analogue scale (VAS). The anchors “not enjoyable at all” and “very enjoyable” were printed at the left and right ends, respectively. Participants were instructed to place an “X” on the line at the point that best represented how much they were enjoying the game at that moment. The distance (mm) from the left anchor to the mark was measured and expressed as a percentage of the total line length, with higher scores indicating greater enjoyment.

#### Flow state

Flow experience during spectating was measured using a brief four-item flow scale adapted from previous research [49]. The scale asked participants the extent to which they (1) forgot the time, (2) were completely focused, (3) lost themselves, and (4) enjoyed themselves while watching the game. Each item was rated on a seven-point Likert scale ranging from 1 (“not at all”) to 7 (“very much”), and the sum of the four items was used as an index of the subjective flow state during spectating. The internal consistency of the scale in this study was acceptable considering the small number of items (Cronbach’s alpha = 0.69).

#### Intention to revisit

To capture behavioral intention to re-engage with live sports spectating, we administered a single-item loyalty scale that assessed intention to revisit: “I would like to watch this event again in the future.” Responses were given on a seven-point Likert scale from 1 (“not at all”) to 7 (“very much”), with higher scores indicating stronger intention to revisit.

#### Sense of unity (Inclusion of Other in the Self)

Perceived social unity with players and fellow spectators was assessed using the Inclusion of Other in the Self (IOS) scale, a widely used pictorial measure of interpersonal closeness [50]. The IOS presents seven pairs of overlapping circles; greater overlap indicates a closer perceived relationship. In this study, participants rated their perceived closeness to four targets: home team players, home team spectators, away team players, and away team spectators. For each target, participants selected the pair of circles that best represented their current sense of unity with that group.

Enjoyment (VAS) and unity (IOS) were assessed at all three time points (pre-game, halftime, post-game) to capture temporal changes across spectating. Flow state and intention to revisit were measured only immediately after the game, reflecting participants’ overall experiential and behavioral responses to the event.

### Statistical analysis

HR synchrony analyses and mediation models were conducted using R statistical software (version 4.4.0; R Foundation for Statistical Computing, Vienna, Austria). All other statistical analyses were performed using GraphPad Prism version 10 (GraphPad Software, San Diego, CA, USA).

To examine oxytocin dynamics, we first employed a linear mixed-effect model (LMM) using baseline oxytocin as a continuous predictor. To further characterize the nonlinear relationship between baseline oxytocin levels and response rates while minimizing inter-individual variability, we classified baseline values into groups in 5 pg/mL increments (averaged within a range of ±2.5 pg/mL) to identify macro-level response trends. Following previous studies, to flexibly capture response dynamics without assuming linearity, we modeled the relationship between these binned baseline levels and post-match change rates using RCS with three knots placed at the 10th, 50th, and 90th percentiles. Subsequently, by analyzing the first derivative (slope) and second derivative (acceleration) of the estimated spline curve, we identified the inflection point (knee point) where the rate of decline reached its maximum value (i.e., the minimum of the second derivative). Based on this data-driven knee point, the cohort was stratified into Low-OT and High-OT groups for visualization and descriptive follow-up analyses. Primary inferential analyses treated baseline oxytocin as a continuous predictor, and no main conclusion depended on this categorical stratification.

Depending on the data structure, group differences and temporal changes were examined using unpaired t-tests or two-way analyses of variance (ANOVA) with factors for group (High-OT vs. Low-OT) and time (pre-game, halftime, post-game). When Mauchly’s test indicated violation of sphericity, Greenhouse–Geisser corrections were applied to the degrees of freedom. When a significant main effect or interaction was detected, Bonferroni’s multiple comparison test was used for post hoc comparisons.

In cases where missing values arose due to incomplete questionnaire responses or exclusion of physiologically implausible outliers, LMM were employed instead of repeated-measures ANOVA to maximize the use of available data. For mixed-effects models, F-statistics and degrees of freedom were used to derive partial η² as an approximate index of effect size. Correlation analyses, for example associations between oxytocin, HR synchrony indices, and psychological measures, were conducted using Pearson’s product–moment correlation coefficient unless otherwise specified.

To verify the robustness of our findings, LMM were also performed for all other primary outcomes, including heart rate synchronization and psychological scores, with baseline oxytocin included as a continuous predictor. These analyses were intended to ensure that the observed effects were not dependent on the specific threshold used for grouping (Supplementary Table 2). In the main analysis, we used an LMM that treated baseline oxytocin levels as a continuous variable. To aid in the interpretation of the results from the continuous model, we present a descriptive visualization showing a plot of the categorical “high oxytocin group/low oxytocin group.”

For mediation analyses, indirect effects were estimated using a nonparametric bootstrapping procedure with 2000 resamples and bias-corrected 95 per cent confidence intervals, implemented in the R package lavaan. An indirect effect was considered statistically significant when the 95 per cent confidence interval did not include zero. All data are presented as mean ± standard deviation (SD) unless stated otherwise, and the threshold for statistical significance was set at p < 0.05 (two-tailed).

## Results

### Salivary oxytocin dynamics shift in a baseline-dependent manner during live sports spectating

To examine whether oxytocin dynamics varied as a function of baseline levels, we first analyzed temporal changes using baseline oxytocin as a continuous predictor. As shown in Fig. 2A and B, individuals with different baseline levels exhibited distinct temporal patterns, suggesting baseline-dependent dynamics. A linear mixed-effects model revealed a significant effect of baseline oxytocin (β = 0.39, t = 4.17, p < 0.0001). Importantly, the interaction between time and baseline oxytocin was significant (F_2, 116_ = 17.72, p < 0.0001, ηp² = 0.23), indicating that the temporal trajectory of oxytocin differed depending on baseline levels.

To further characterize this relationship, we examined the association between baseline oxytocin and subsequent oxytocin responses using a spline model. For visualization, baseline values were grouped into bins (5-unit intervals, averaged within ±2.5 ranges), and the mean response was plotted (Fig. 2C). The spline analysis suggested a nonlinear relationship, with a clear inflection point (knee point) observed at approximately 26.5 pg/ml (Supplementary Fig. 1). This pattern indicates that oxytocin dynamics may differ above and below this threshold. For descriptive visualization, participants were stratified into Low-OT and High-OT groups based on this threshold. However, baseline oxytocin was treated as a continuous predictor in the primary LMMs, and no main conclusion depended on this threshold (Supplementary Table 2).

Examining dynamics within each group after classification, the High-OT group maintained elevated oxytocin concentrations throughout the game, showing no evidence of further time-dependent change, whereas the Low-OT group exhibited a significant increase over time that brought post-game oxytocin levels into the range observed in the High-OT group (High-OT main group effect: F_1, 58_ = 28.40, p < 0.0001, ηp² = 0.33; Low-OT time effect: F_1.95, 113.6_ = 9.92, p = 0.0001, ηp² = 0.15; Fig. 2D). Although this descriptive group comparison could partly reflect statistical regression to the mean, the observed temporal pattern was consistent with baseline-dependent salivary oxytocin dynamics during spectatorship. Cortisol concentrations decreased across both groups during spectating (F_1.78, 103.1_ = 3.32, p = 0.045, ηp² = 0.05; Fig. 2E), resulting in a progressive increase in the oxytocin-to-cortisol ratio (F_1.63, 94.74_ = 14.50, p < 0.0001, ηp² = 0.20; Fig. 2F). These patterns in cortisol and oxytocin-to-cortisol ratio were also confirmed using LMM with baseline oxytocin as a continuous predictor, yielding consistent results. Similar stability was observed for heart rate synchrony and psychological scores, where the continuous and categorical models showed no significant deviations in the overall findings. Taken together, these findings indicate that sports spectating may modulate hormonal dynamics in a baseline-dependent manner, sustaining high oxytocin levels in one subgroup and augmenting them in another, together with increased oxytocin-to-cortisol ratios and no evidence of cortisol elevation.

### High oxytocin is linked to unity through increased enjoyment

**Fig. 3:**
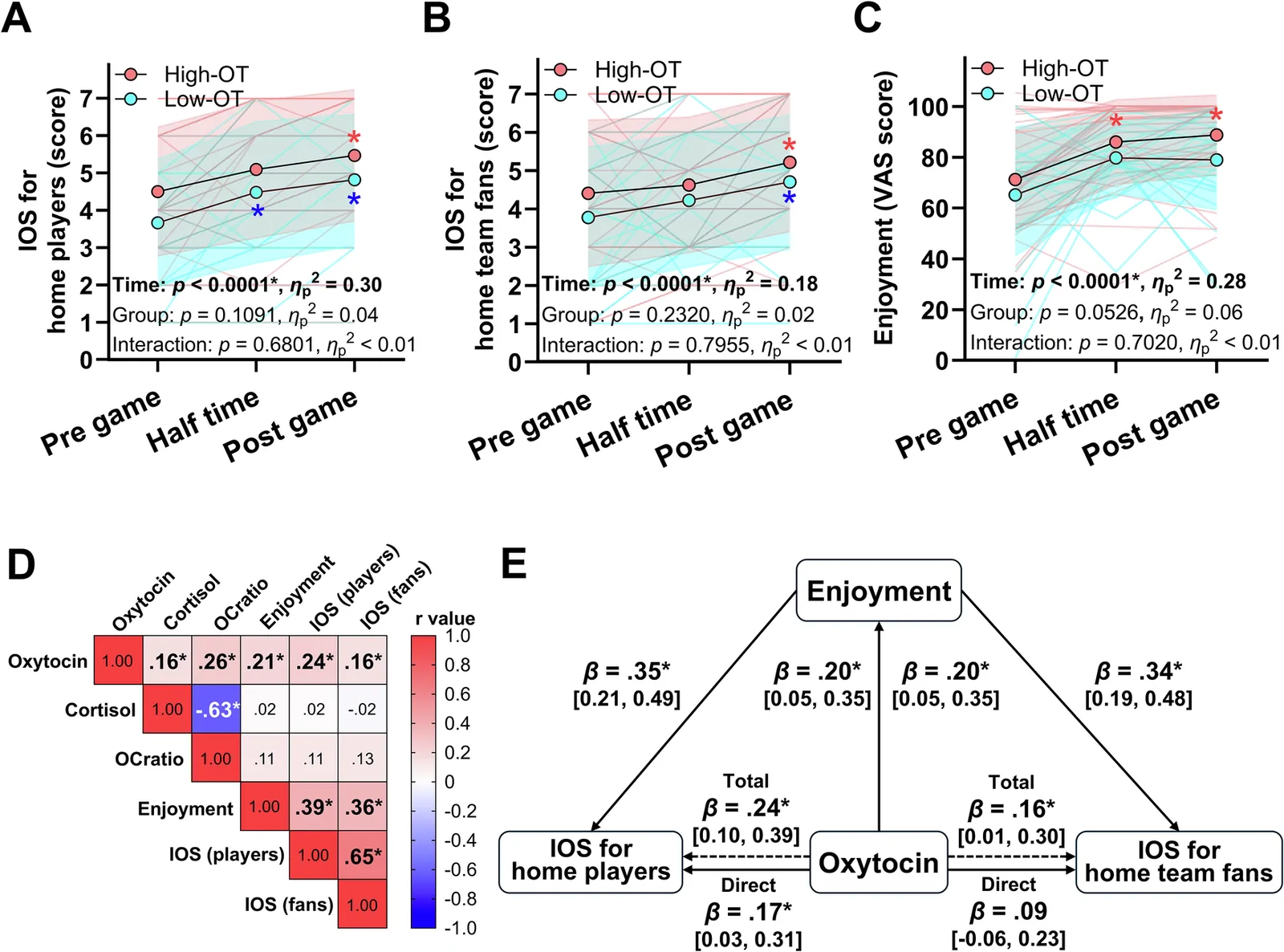
High oxytocin is linked to unity through increased enjoyment. **A** Temporal changes in perceived unity for home players. **B** Unity for home team fans. **C** Enjoyment. The High-OT group is shown in red (n = 32) and Low-OT group is shown in blue (n = 27). Data points represent group means, with shaded bands representing the standard deviation (SD) and thin lines representing individual trajectories. Results of two-way mixed-model ANOVA (factors: Time, Group, and Interaction) are indicated in the lower-left corner of each panel. Post-hoc comparisons were performed using Bonferroni’s multiple comparison test: *p < 0.05 vs. pre-game for both groups; # p < 0.05 vs. Low-OT group at the same point. **D** Correlation matrix between neuroendocrine responses, mood and a sense of unity (n = 59). The significance of the correlation analysis is indicated at the top-right of correlation coefficient. *p < 0.05. **E** Mediation model showing the associations between oxytocin, enjoyment, and a sense of unity (n = 59). Standardized mediation coefficients (β) and their bootstrap 95% confidence intervals in brackets are shown for all pathways. Path coefficients from separate mediation analyses for unity with players (left) and fans (right) are presented. *p < 0.05. The reduced sample size was due to one missing questionnaire response. The High-OT and Low-OT grouping is shown for descriptive visualization only; primary analyses treated baseline oxytocin as a continuous predictor.

We next examined how oxytocin dynamics during spectating relate to subjective experiences, focusing on social bonding and enjoyment. Unity with the home team players and home team fans increased over time irrespective of baseline oxytocin group (players: F_1.48, 84.59_ = 24.68, p < 0.0001, ηp² = 0.30; fans: F_1.83, 104.2_ = 12.38, p < 0.0001, ηp² = 0.18; Fig. 3A, B). Enjoyment also increased in both groups (F_1.78, 101.5_ = 21.86, p < 0.0001, ηp² = 0.28; Fig. 3C).

Across all time points, higher oxytocin concentrations were positively associated with greater enjoyment (r = 0.21, p = 0.006, 95% CI [0.06, 0.34]), stronger unity with players (r = 0.24, p = 0.001, 95% CI [0.10, 0.38]), and stronger unity with fans (r = 0.16, p = 0.037, 95% CI [0.001, 0.30]; Fig. 3D). Mediation analyses further showed that the association between oxytocin and perceived unity with players was accounted for by increased enjoyment (indirect effect: z = 2.74, p = 0.006, 95% CI [0.005, 0.023]; Fig. 3E), and similarly unity with fans through enjoyment (indirect effect: z = 2.61, p = 0.009, 95% CI [0.004, 0.022]; Fig. 3E). These results suggest that baseline-dependent salivary oxytocin dynamics may be involved in enjoyment-linked unity during spectating, highlighting enjoyment as a statistical link between oxytocin levels and perceived social bonding among spectators.

### HR dynamics and its synchrony analysis condition

**Fig. 4:**
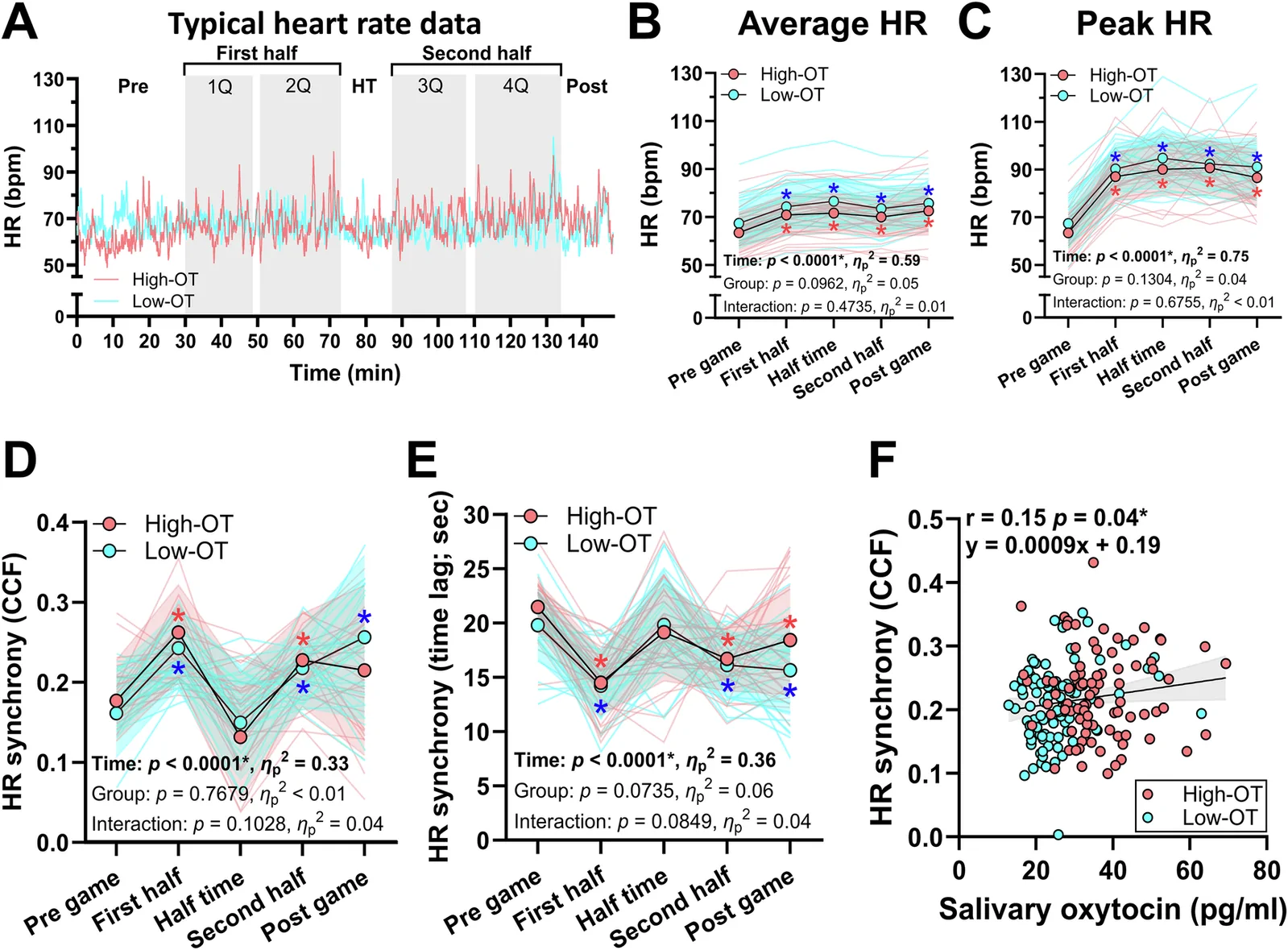
Salivary oxytocin dynamics co-occur with HR synchrony. **A** Typical HR data. **B** Temporal changes in average HR. **C** Peak HR. **D** Similarity of HR dynamics (cross-correlation function: CCF). **E** Time lag. The High-OT group is shown in red (n = 32) and Low-OT group is shown in blue (n = 27). Data points represent group means, with shaded bands representing the standard deviation (SD) and thin lines representing individual trajectories. Results of two-way mixed-model ANOVA (factors: Time, Group, and Interaction) are indicated in the lower-left corner of each panel. Post-hoc comparisons were performed using Bonferroni’s multiple comparison test: *p < 0.05 vs. pre-game for both groups. **F** Correlation between oxytocin and HR synchrony (n = 59). Pearson’s product-moment correlation coefficient and the results of the single regression analysis are shown in the upper-left corner of each graph. The computed regression line and 95% confidence intervals are shown as bands on the graph. *p < 0.05. The reduced sample size was due to one missing HR recording. The High-OT and Low-OT grouping is shown for descriptive visualization only; primary analyses treated baseline oxytocin as a continuous predictor.

We then investigated whether live sports spectating increases HR synchrony among spectators and whether this synchrony is associated with oxytocin. Mean HR and peak HR followed similar trajectories in the High-OT and Low-OT groups, with both indices showing robust elevations relative to pre-game levels (mean HR: F_2.54, 145.1_ = 83.00, p < 0.0001, ηp² = 0.59; peak HR: F_3.13, 178.6_ = 169.2, p < 0.0001, ηp² = 0.75; Fig. 4B, C).

Next, we examined the degree of heart rate synchrony among spectators. Before conducting the main heart rate synchrony analysis, we examined whether the estimated synchronization depended on the choice of temporal lag window. We transformed the correlation coefficients between the heart rate synchrony indices calculated under multiple lag window conditions and the subjective indices and compared their total values. As a result, heart rate synchronization calculated using a 30-second lag window showed the strongest association with the subjective indices (In-game: F_1.36, 14.90_ = 28.81, p < 0.0001, η^2^ = 0.38; Out of game: F_1.74, 19.19_ = 14.11, p = 0.0003, η^2^ = 0.42; Supplementary Fig. 2).

### Salivary oxytocin dynamics co-occur with HR synchrony

Cross-correlation analyses using this 30 s lag window revealed that HR synchrony, indexed by CCF values, increased significantly during the first and second halves of the game, with no baseline differences in HR synchrony between the High-OT and Low-OT groups (F_2.98, 170.0_ = 27.74, p < 0.0001, ηp² = 0.33; Fig. 4D). In parallel, the temporal lag between HR time series shortened during and after the game (F_3.07, 175.0_ = 31.65, p < 0.0001, ηp² = 0.36; Fig. 4E), indicating tighter moment-to-moment alignment of cardiac dynamics.

Importantly, to verify that the observed HR synchrony reflects context-specific shared engagement rather than merely shared stimulus-driven covariance, we examined surrogate pairings. Synchrony between participants attending the same game (intra-game) was significantly higher than pseudo-synchrony calculated between participants attending different games (inter-game) (Supplementary Fig. 3). Similarly, synchrony was stronger among individuals seated in the same block (intra-block) compared to those in different blocks (inter-block) (Supplementary Fig. 4). These control analyses suggest that the observed physiological alignment was context-specific and unlikely to reflect nonspecific covariance alone, although shared local context and game-related stimulus locking likely contributed.

Oxytocin concentrations were positively related to HR synchrony (r = 0.15, p = 0.041, 95% CI [0.006, 0.294]; Fig. 4F). Thus, we interpret this association as reflecting salivary oxytocin levels during spectatorship rather than evidence that increases in oxytocin from baseline drove HR synchrony. This pattern suggests that the absolute level of salivary oxytocin during spectatorship, rather than the magnitude of change from baseline alone, may be more relevant to its association with physiological alignment. This association was especially pronounced in the Low-OT group, in which oxytocin levels were significantly associated with both CCF values (r = 0.28, p = 0.009, 95% CI [0.07, 0.46]) and time-lag indices (r = −0.26, p = 0.014, 95% CI [−0.44, −0.05]; Supplementary Fig. 7B&D). These findings suggest that HR synchrony increases among casual spectators regardless of baseline oxytocin level, and that salivary oxytocin levels, particularly in individuals with lower baseline concentrations, were associated with stronger physiological alignment during spectating. Importantly, these effects were observed in modestly sized collegiate games, indicating that synchrony-related associations with social connection may also be observed outside highly fused fan groups typical of professional settings.

Furthermore, we confirmed that these overall patterns of baseline-dependent oxytocin dynamics and HR synchrony were generally consistent regardless of participant sex (Supplementary Fig. 5) or which of the two games was attended (Supplementary Fig. 6).

### HR synchrony relates to the spectating experience through a sense of unity and loyalty

**Fig. 5:**
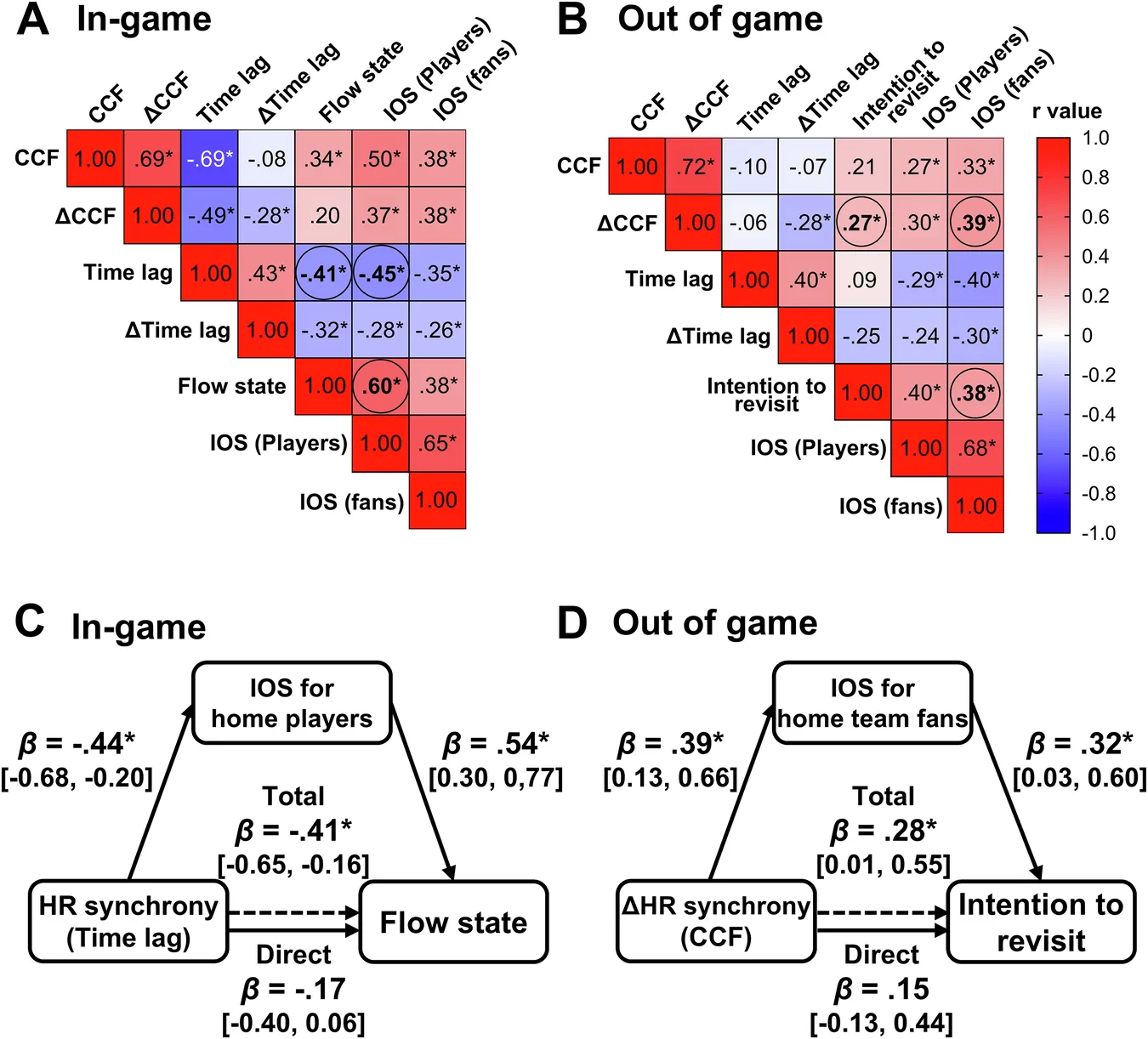
HR synchrony relates to the spectating experience through a sense of unity and loyalty. **A** Correlation matrix between measurements related to in-game (n = 58–59). **B** Correlation matrix between measurements related to out of game (n = 53–59). The significance of the correlation analysis is indicated at the top-right of correlation coefficient. *p < 0.05. **C** Mediation model of in-game (n = 58). **D** Mediation model of out of game (n = 53). Standardized mediation coefficients (β) and their bootstrap 95% confidence intervals in brackets are shown for all pathways. *p < 0.05. Sample sizes varied by model: n = 58 for models including one missing questionnaire response and one missing HR recording; n = 53 for models including six missing questionnaire responses and one missing HR recording.

Finally, we examined how physiological synchrony relates to social bonding and the overall spectating experience, in line with the hypothesis that HR synchrony would support psychological engagement via unity. To characterize the functional role of synchrony, HR data were reclassified into in-game periods (active play) and out-of-game periods (inter-quarter intervals, halftime, and post-game). During in-game periods (Fig. 5A), shorter time-lag values between HR time series were significantly associated with stronger unity toward home team players (r = −0.44, p < 0.001, 95% CI [−0.63, −0.20]) and higher flow state (r = −0.41, p = 0.001, 95% CI [−0.60, −0.17]), indicating that tighter moment-to-moment alignment of cardiac dynamics was linked to more affiliative and immersive responses. During out-of-game periods (Fig. 5B), increases in CCF relative to pre-game (ΔCCF) were positively correlated with unity toward home spectators (r = 0.38, p = 0.003, 95% CI [0.13, 0.58]) and with loyalty to the team, indexed by intention to revisit (r = 0.28, p = 0.043, 95% CI [0.01, 0.51]), suggesting that synchrony outside active play reflects broader social bonding among spectators.

To test whether social bonding mediated these relationships, we conducted mediation analyses. In-game, HR synchrony, indexed by shorter time lag, is associated with flow state via unity with players as a mediator (z = −3.32, p < 0.001, 95% CI [−0.69, −0.19]; Fig. 5C). Out-of-game, ΔCCF showed a significant indirect association with team loyalty via unity with fellow fans (z = 2.01, p = 0.018, 95% CI [0.52, 4.83]; Fig. 5D). These results suggest that physiological synchrony was associated with social-bonding-related experiences, which in turn were linked to immersive flow and loyalty to the team during live spectating, consistent with our hypothesis that oxytocin-related dynamics, HR synchrony, and social connection would covary during live spectating.

## Discussion

Live sports spectating in a modest collegiate setting suggests that an everyday collective experience is associated with salivary oxytocin dynamics and context-specific physiological alignment linked to social connection among casual spectators. Oxytocin showed baseline-dependent dynamics together with an endocrine profile characterized by decreased cortisol and an increased oxytocin-to-cortisol ratio (Fig. 2D–F), and—critically—oxytocin levels covaried with interpersonal HR synchrony during the game (Fig. 4F). Given the modest and heterogeneous benefits of exogenous intranasal oxytocin in clinical trials [5–9, 11, 57], this oxytocin–synchrony coupling motivates a complementary translational hypothesis: scalable, non-pharmacological collective contexts may engage endogenous oxytocin-linked systems in measurable ways that relate to unity-relevant outcomes.

Below, we interpret the endocrine profile (Section 4.1), outline candidate mechanisms (Section 4.2), summarize phase-specific synchrony–experience associations (Section 4.3), and then focus on the oxytocin–synchrony coupling and translational implications (Sections 4.4–4.5), before noting limitations and future directions (Section 4.6).

### Baseline-dependent oxytocin dynamics and hormonal profile

Sports spectating is associated with oxytocin dynamics in a baseline-dependent manner: spectators with higher pre-game oxytocin maintained elevated concentrations across the event, whereas those with lower baseline oxytocin showed marked increases that brought post-game levels into a similar range (Fig. 2A; Supplementary Table 1). Across both groups, cortisol decreased and the oxytocin-to-cortisol ratio increased (Fig. 2B, C). Although the cortisol decrease may partly reflect the natural circadian decline associated with the late-day timing of the games, the absence of cortisol elevation, together with the increased oxytocin-to-cortisol ratio, is consistent with a relatively oxytocin-dominant rather than stress-dominated endocrine profile during spectatorship.

A plausible interpretation is that live spectatorship provides an affiliative, narrative-rich social context—shared attention to an emotionally salient, unfolding contest and coordinated collective reactions—that can support endogenous oxytocin dynamics without pharmacological manipulation [51, 52]. The collective enjoyment of an unscripted contest may function as a form of “embodied storytelling” that amplifies shared affect and togetherness. Consistent with this view, higher oxytocin levels were associated with greater enjoyment and stronger perceived unity, and mediation analyses suggested that enjoyment partially accounted for oxytocin–unity associations (Fig. 3E). This endocrine profile provides context for examining how endocrine state relates to interpersonal physiological coupling during spectatorship (Section 4.4).

### Theoretical candidate neural mechanisms linking oxytocin, shared experience, and physiological synchrony

What neurobiological processes might connect live spectatorship to coordinated endocrine and autonomic dynamics? One plausible account is that collective spectatorship aligns attention, affect, and prediction across individuals via shared sensory input and a common unfolding narrative, engaging neural systems for salience detection, reward, and social appraisal [29, 30]. Live sports events provide sustained shared attention, rapid shifts in uncertainty (e.g., scoring opportunities), and collective affective reactions, which can recruit salience-related circuitry and reward/motivation systems (e.g., ventral striatum and medial prefrontal regions) [53–56]. Such shared, time-locked processing is expected to shape autonomic output, yielding temporally aligned HR fluctuations across spectators who attend to the same event structure and appraise it in similar ways [30, 57].

Theoretically, oxytocin may influence these shared-experience processes through two complementary routes. First, oxytocin is thought to tune the salience and rewarding value of socially meaningful cues and affiliative contexts, interacting with mesolimbic and limbic–prefrontal circuitry to bias attention and motivation toward social stimuli [3, 19, 58, 59]. In a crowd setting, such tuning could amplify convergence in attentional focus and affective appraisal—conditions that favour physiological alignment—without requiring explicit dyadic interaction [30, 57]. Second, oxytocin is implicated in stress buffering and autonomic regulation [19]; thus, an oxytocin-dominant endocrine balance without cortisol elevation (Fig. 2E, F) is consistent with a milieu in which social engagement can unfold without pronounced stress-related endocrine costs. This may help explain why modest collegiate spectatorship can be associated with increased unity and positive experiential outcomes while avoiding the pronounced cortisol elevations reported in highly invested fans and high-stakes contexts [60]. These candidate mechanisms motivate our subsequent focus on how endocrine state relates to interpersonal physiological coupling and unity-related outcomes in this naturalistic setting.

### Physiological synchrony, unity, and phase-specific links to experiential outcomes

Interpersonal physiological synchrony in groups can arise from shared sensory input and event boundaries, but it is also shaped by convergent appraisal, shared affect, and affiliation—processes that are central to collective bonding [29–32, 57]. In the present field setting, HR synchrony increased during live spectating and showed phase-specific associations with subjective and motivational outcomes. Specifically, stronger synchrony was associated with greater perceived unity, higher flow, and stronger intention to revisit/loyalty, with distinct patterns during the game versus outside active play (Figs. 4D, E, 5A, B). These findings are consistent with the view that synchrony indexes the extent to which spectators share attention and affect in socially meaningful ways, beyond mere co-exposure to the same stimuli.

Mediation models further suggested that perceived unity statistically links synchrony to downstream experiential and motivational outcomes (Fig. 5C, D). While these analyses do not establish causal direction, they support a coherent interpretation in which momentary alignment of autonomic dynamics during shared events covaries with unity-related appraisals that, in turn, relate to positive experiential states and the motivation to re-engage. This phase-resolved synchrony–experience pattern provides the immediate context for our focus in Section 4.4 on whether, and how, endogenous oxytocin covaries with synchrony as a coupled socio-physiological signature of shared engagement.

### Oxytocin–synchrony coupling as a socio-physiological signature of shared engagement

A central observation of the present study is that endogenous oxytocin levels positively covaried with interpersonal HR synchrony during live spectatorship (Fig. 4F). This initial finding links endocrine state to interpersonal alignment of autonomic dynamics in a naturalistic crowd setting and extends prior work showing that physiological synchrony in dyads and groups is associated with affiliation, shared affect, and social connection [30–32, 57]. The overall oxytocin–HR synchrony association was small (r = 0.15) and therefore should not be interpreted as a strong individual-level predictor. Because HR synchrony increased in both baseline-oxytocin groups, this association is best interpreted in relation to salivary oxytocin levels during spectatorship rather than as evidence that increases from baseline drove synchrony. We instead view this effect as a modest association within a noisy naturalistic field setting, whose relevance lies in its convergence with baseline-dependent salivary oxytocin dynamics, increased HR synchrony during the game, and synchrony–unity associations. In our data, the oxytocin–synchrony association co-occurred with (i) an endocrine profile characterized by an increased oxytocin-to-cortisol ratio without cortisol elevation (Fig. 2E&F), and (ii) phase-specific associations in which stronger synchrony related to greater unity, higher flow, and stronger intention to revisit (Figs. 4D, E, 5A, B). Together, these convergent patterns are consistent with the idea that oxytocin dynamics and interpersonal physiological coupling jointly index the extent to which spectators share attention and affect in socially meaningful ways during a live event.

At least two non-mutually-exclusive mechanisms could account for this oxytocin–synchrony coupling. Consistent with the mechanism outlined above, oxytocin may be associated with synchrony through its putative role in social salience and reward valuation via mesolimbic and limbic–prefrontal circuits, thereby biasing attention and affective appraisal toward shared, socially meaningful cues in the crowd [3, 19, 58, 59, 61]. Such tuning could promote convergence in spectators’ responses—and thus tighter temporal alignment of autonomic dynamics—without requiring explicit dyadic interaction [30, 57]. These candidate accounts are compatible with the observed positive associations between oxytocin, enjoyment, and unity (Fig. 3C–E) and with models suggesting that enjoyment and perceived unity statistically link socio-physiological responses to experiential outcomes (Figs. 3E, 5C, D).

A key alternative explanation is that both oxytocin and HR synchrony may be driven by common external inputs or generalized arousal. Synchrony in groups can arise from shared event boundaries, common sensory stimulation, and parallel attentional orienting, all of which can produce correlated autonomic fluctuations even in the absence of interpersonal co-regulation [30, 57]. Likewise, emotionally salient moments in a game could simultaneously influence endocrine and autonomic responses. However, the present pattern suggests that synchrony was unlikely to reflect nonspecific co-exposure alone: stronger synchrony related to unity and downstream experiential/motivational outcomes in a phase-dependent manner (Figs. 4D, E, 5A, B), and unity statistically mediated associations between synchrony and flow or intention to revisit (Fig. 5C, D). These findings are consistent with the view that convergent appraisal and social meaning—rather than stimulus exposure alone—contribute to synchrony–experience links in this context [29–32]. Future studies can strengthen causal inference by combining the present field approach with (i) control analyses that index “pseudo-synchrony” using shuffled pairings or time-shifted series [30, 57], (ii) covariate adjustment for average HR or other arousal proxies, and (iii) designs that manipulate shared appraisal (e.g., unified vs divided attention, varying social framing, or spectator interaction opportunities).

From a translational perspective, the oxytocin–synchrony coupling observed here (Fig. 4F) nominates interpersonal physiological synchrony as a potentially scalable, non-invasive readout of endogenous oxytocin-linked social engagement in everyday settings. Whereas exogenous oxytocin administration faces challenges related to efficacy heterogeneity, dosing, and long-term safety [6–9, 11, 62, 63], naturalistic collective experiences may engage oxytocin-linked systems through contextualized shared attention, affect, and meaning. In this framework, live sports spectatorship offers a practical platform for developing and testing low-burden strategies that scaffold social connection, with oxytocin dynamics and physiological synchrony serving as complementary candidate biomarkers of engagement and unity-related outcomes.

### Clinical and translational implications

Social disconnection and impaired social functioning are transdiagnostic challenges in psychiatry. Although oxytocin has been pursued as a pharmacological target for social dysfunction, multisite and longer-term trials of intranasal oxytocin show null-to-modest and heterogeneous effects with practical uncertainties regarding dosing and long-term safety [6–9, 11, 62, 63]. This motivates scalable, non-pharmacological contexts that engage endogenous oxytocin-linked systems in everyday life.

Here, live sports spectatorship was associated with baseline-dependent oxytocin dynamics and an endocrine profile characterized by an increased oxytocin-to-cortisol ratio without cortisol elevation (Fig. 2D–F), and oxytocin covaried with interpersonal HR synchrony during the game (Fig. 4F). This oxytocin–synchrony signature related to unity-relevant outcomes (enjoyment, perceived unity) and downstream experiential/motivational outcomes (flow and intention to revisit; Figs. 3C–E, 4D, E, 5A–D), with mediation analyses indicating that enjoyment and unity statistically linked socio-physiological responses to outcomes (Figs. 3E, 5C, D). These observations complement social-psychological models of fandom [35] and align with community evidence linking live spectatorship and loyalty with social support and reduced loneliness [36, 37]. Together, the results highlight the potential of live sports spectatorship as a practical, scalable non-pharmacological context characterized by an oxytocin–synchrony signature of shared social engagement in everyday life.

Importantly, the core ingredients of this setting—shared attention, collective emotional dynamics, and opportunities for affiliative exchange—are not limited to traditional sport venues. Similar collective viewing contexts are rapidly expanding in e-sports arenas and large-scale live-streaming events, where design features that enhance social presence (including the sharing of biosignals) may further scaffold interpersonal engagement [64]. Testing whether comparable oxytocin–synchrony signatures emerge in these hybrid physical–digital settings may help bridge social neuroscience with scalable interventions for loneliness and social dysfunction.

### Limitations and future directions

Several limitations should be considered when interpreting these findings. First, the study was observational and did not include a non-spectating control condition, which limits causal inference about live spectatorship per se. Unmeasured situational factors, including event-specific contextual features from the two games studied (e.g., performance expectations, game outcome, score progression, crowd atmosphere, co-attendee relationships, pre-existing team loyalty, and moment-to-moment game dynamics) may have contributed to endocrine, synchrony, and experiential responses. Controlled or quasi-experimental comparisons (e.g., live vs. at-home viewing, non-social entertainment, or attendance with different social framing) and longitudinal follow-up will therefore be important for testing whether the oxytocin–synchrony signature predicts durable changes in social-connection-relevant outcomes. Because this study was not preregistered, and because multiple outcomes and analytical choices were involved, the findings should be interpreted as exploratory and hypothesis-generating rather than strictly confirmatory. We nevertheless sought to improve transparency by reporting the a priori power analysis, statistical models, grouping rationale, correction methods, and control analyses in detail.

Second, baseline oxytocin showed marked inter-individual variability, and our knee point-based stratification should be interpreted as a descriptive approach to capturing heterogeneity. Although this grouping was not attributable to physical characteristics such as body composition, resting HR, or habitual physical activity (Supplementary Table 1), baseline variability may reflect stable traits or transient influences (e.g., recent social interactions). While LMM using baseline oxytocin as a continuous variable supported the robustness of the overall findings (Supplementary Table 2), this grouping was retained only to provide a clearer visualization of how baseline levels may shape the increase or maintenance of oxytocin dynamics.

Third, endocrine interpretation is constrained by the nature and timing of the hormone measurements. Oxytocin was measured in saliva, and inference to central oxytocin dynamics remains indirect amid ongoing debate about central–peripheral coupling [65–68]. Stronger mechanistic inference will require convergent approaches, such as validated peripheral sampling combined with neuroimaging and, where feasible, experimental stimulation or pharmacological challenges [12]. In addition, because cortisol follows a strong circadian rhythm, the observed decrease in cortisol may partly reflect late-day circadian decline rather than event-specific effects. To characterize endocrine dynamics as much as possible while preserving a natural viewing environment, saliva samples were collected at three feasible time points: pre-game, halftime, and post-game. Nevertheless, this sampling schedule may not have been sufficient to precisely identify the onset, peak, or duration of oxytocin and cortisol responses. Individual hormonal status, such as menstrual cycle phase or hormonal contraception use, was also not controlled in order to preserve ecological validity, potentially introducing unmeasured variance into the endocrine data. Future studies should record these factors and examine their potential influence on individual neuroendocrine responses.

Fourth, the observed oxytocin–HR synchrony covariation could partly reflect shared external drivers or generalized arousal. Although we included pseudo-synchrony analyses using inter-game and inter-block comparisons, shared game-related stimulus locking and local viewing context likely contributed to the observed physiological alignment. Future studies should extend these controls by incorporating time-shifted series, adjustment for arousal proxies, and designs that manipulate shared appraisal or social framing.

Finally, generalizability remains to be established. Our synchrony analyses focused on spectator dyads with relatively similar attributes and backgrounds, drawn mostly from young adults attending modest collegiate games. Whether similar oxytocin–synchrony associations are observed across different demographics, cultural contexts, highly identified fan populations, high-stakes professional environments, and other interaction targets (e.g., spectator–player synchrony) remains an open question. Addressing these issues in larger and more diverse samples will be essential for evaluating boundary conditions, feasibility, and equity of access, particularly when extending this approach to populations characterized by elevated social disconnection or social dysfunction.

Despite these limitations, the present field data provide an ecologically valid starting point for translational work examining how widely accessible collective experiences are associated with salivary oxytocin dynamics, interpersonal physiological coupling, and social connection in everyday life.

## Conclusion

In a real-world collegiate setting, a single session of live sports spectatorship was associated with coordinated endogenous oxytocin dynamics and interpersonal HR synchrony, with oxytocin levels showing a slight but positive association with synchrony during the game. This coupled socio-physiological signature was linked to social-connection–relevant outcomes, including enjoyment, perceived unity with players and fellow fans, and downstream experiential and motivational indices (flow and intention to revisit). Together, these findings position live sports spectatorship as a practical, scalable non-pharmacological context that is characterized by an oxytocin–synchrony signature of shared social engagement in everyday life.

## Supplementary information

Supplementary Material

## Data Availability

The data supporting the findings of this study are available from the corresponding author upon reasonable request.
