# Supplementary material for: Endogenous oxytocin-linked heart-rate synchrony and social connection during live sports spectatorship
Source: Transl Psychiatry. 2026 Jul 8;16:517. doi: 10.1038/s41398-026-04265-2 (PMC13635218; doi:10.1038/s41398-026-04265-2)
Supplement: Supplementary file 1 [file 41398_2026_4265_MOESM1_ESM.docx]

**Supplementary Table 1 Participants’ characteristics**

|  | **High-OT**  **(n = 33)** | | | **Low-OT**  **(n = 27)** | | | ***p*** | ***d*** |
| --- | --- | --- | --- | --- | --- | --- | --- | --- |
| Knee point (oxytocin; pg/ml) | 26.5 | | | | | |  |  |
| Baseline oxytocin levels (pg/ml) | 38.0 | ± | 9.5^#^ | 21.0 | ± | 3.4 | <0.01 | 2.31 |
| Age (years) | 22.1 | ± | 8.3 | 23.1 | ± | 9.3 | 0.68 | 0.11 |
| Gender (n, Male/Female) | 23 | / | 10 | 16 | / | 11 |  |  |
| Height (cm) | 171.9 | ± | 9.7 | 168.9 | ± | 7.4 | 0.21 | 0.34 |
| Body weight (kg) | 68.2 | ± | 15.8 | 65.6 | ± | 8.4 | 0.46 | 0.20 |
| BMI (kg/m^2^) | 22.8 | ± | 3.5 | 22.9 | ± | 1.9 | 0.90 | 0.03 |
| Resting HR (bpm) | 63.4 | ± | 8.5 | 67.3 | ± | 9.2 | 0.09 | 0.44 |
| Vigorous-intensity activity  (min/day) | 85.0 | ± | 81.4 | 83.1 | ± | 82.3 | 0.93 | 0.02 |
| Moderate-intensity activity  (min/day) | 22.6 | ± | 34.5 | 10.3 | ± | 17.8 | 0.13 | 0.42 |
| Walking time (min/day) | 24.3 | ± | 29.2 | 28.1 | ± | 41.4 | 0.69 | 0.11 |
| Exercise time (min/day) | 70.4 | ± | 68.4 | 70.0 | ± | 83.8 | 0.98 | <0.01 |
| Sitting time (min/day) | 345.5 | ± | 221.4 | 283.3 | ± | 163.7 | 0.24 | 0.31 |
| Cheering for home team (score) | 6.5 | ± | 0.7 | 6.3 | ± | 0.9 | 0.31 | 0.27 |

Values are presented as mean ± SD. #: p < 0.05 vs. Low-OT.

**Supplementary Table 2 Results summary of linear mixed model.**

| Item | Effect | F value | P value | Effect size (*η*_p_^2^) |
| --- | --- | --- | --- | --- |
| Cortisol | Time | 3.34 | 0.04 | 0.05 |
|  | Baseline OT | 2.77 | 0.10 | 0.05 |
|  | Interaction | 0.68 | 0.51 | 0.01 |
| Oxytocin/Cortisol ratio | Time | 14.36 | <0.01 | 0.20 |
|  | Baseline OT | 1.66 | 0.20 | 0.03 |
|  | Interaction | 0.73 | 0.49 | 0.01 |
| IOS for  home players | Time | 24.34 | <0.001 | 0.30 |
|  | Baseline OT | 4.90 | 0.03 | 0.08 |
|  | Interaction | 0.02 | 0.98 | <0.01 |
| IOS for  home team fans | Time | 12.37 | <0.01 | 0.18 |
|  | Baseline OT | 3.01 | 0.09 | 0.05 |
|  | Interaction | 0.25 | 0.78 | <0.01 |
| Enjoyment | Time | 22.16 | <0.01 | 0.28 |
|  | Baseline OT | 4.09 | 0.05 | 0.07 |
|  | Interaction | 0.03 | 0.97 | <0.01 |
| Average HR | Time | 83.21 | <0.01 | 0.59 |
|  | Baseline OT | 3.14 | 0.08 | 0.05 |
|  | Interaction | 0.46 | 0.76 | <0.01 |
| Peak HR | Time | 171.27 | <0.01 | 0.75 |
|  | Baseline OT | 1.49 | 0.23 | 0.03 |
|  | Interaction | 0.62 | 0.65 | 0.01 |
| CCF | Time | 26.68 | <0.01 | 0.32 |
|  | Baseline OT | 0.29 | 0.59 | <0.01 |
|  | Interaction | 1.45 | 0.22 | 0.02 |
| Time lag | Time | 30.97 | <0.01 | 0.35 |
|  | Baseline OT | 0.07 | 0.79 | <0.01 |
|  | Interaction | 0.60 | 0.66 | 0.01 |

**Supplementary Figure 1 Identification of the knee point using spline-derived slope and acceleration.**

The blue curve shows the first derivative (slope) and the red curve shows the second derivative (acceleration) of the restricted cubic spline model relating baseline salivary oxytocin to the post-game oxytocin response rate. The knee point was defined as the baseline oxytocin value where the acceleration reached its minimum, indicating the point of maximal change in the response trajectory.

**Supplementary Figure 2 Sensitivity analysis of time-lag parameters in heart rate synchrony.**

**A.** Correlation matrices between synchrony and psychological measurements across different lag windows (10, 30, and 60 s from left to right) of in-game. **B.** Out of game. The significance of the correlation analysis is indicated at the top-right of correlation coefficient. * p < 0.05. **C.** Mean Fisher z-transformed correlation coefficients across lag conditions for in-game. **D.** Out of game.

**Supplementary** **Figure 3 Within and between-game heart rate synchrony across two games.**

**A.** Similarity of HR dynamics for Intra-game (n = 59, light blue) and Inter-game (n = 59, gray). **B.** Similarity of HR dynamics across participants for the High-OT (n = 32, red) and Low-OT (n = 27, blue) groups. **C.** Time lag across all participants. **D.** Time lag across all participants for the High-OT and Low-OT groups. Data points represent group means, with shaded areas indicating standard deviation (SD), and thin lines representing individual trajectories. Statistical results from two-way mixed-model ANOVA (factors: Time, Group and Interaction) are shown in A and C, whereas B and D display results from three-way mixed-model ANOVA (factors: Intra vs. Inter and High vs. Low). Post-hoc comparisons were conducted using Bonferroni’s correction: # p < 0.05 vs. Inter-game at the same point.

**Supplementary Figure 4 Within- and between-block heart rate synchrony across two seating blocks.**

**A.** Similarity of HR dynamics for Intra-block (n = 59, light blue) and Inter-block (n = 59, gray). **B.** Similarity of HR dynamics across all participants for the High-OT (n = 32, red) and Low-OT (n = 27, blue) groups. **C.** Time lag for Intra-block and Inter-block. **D.** Time lag for the High-OT and Low-OT groups. Data points represent group means, with shaded areas indicating standard deviation (SD), and thin lines representing individual trajectories. Statistical results from two-way mixed-model ANOVA (factors: Time, Group and Interaction) are shown in A and C, whereas B and D display results from three-way mixed-model ANOVA (factors: Intra vs. Inter and High vs. Low). Post-hoc comparisons were conducted using Bonferroni’s correction.

**Supplementary Figure 5 Sex differences in temporal changes of oxytocin and heart rate synchrony.**

**A.** Temporal changes in salivary oxytocin. **B.** Similarity of HR dynamics. **C.** Time lag. High-OT group is shown in the left panels, and Low-OT group is shown in the right panels. Data points represent group means, with shaded bands representing the standard deviation (SD) and thin lines representing individual trajectories. Results of two-way mixed-model ANOVA (factors: Time, Group, and Interaction) are indicated in the lower-left corner of each panel. Post-hoc comparisons were performed using Bonferroni’s multiple comparison test: *p < 0.05 vs. pre-game for both groups; # p < 0.05 vs. Female group at the same point.

**Supplementary Figure 6 Game differences in temporal changes of oxytocin and heart rate synchrony.**

**A.** Temporal changes in salivary oxytocin. **B.** Similarity of HR dynamics. **C.** Time lag. Data of High-OT group is shown in the left panels, and the Low-OT group is shown in the right panels. Data points represent group means, with shaded bands representing the standard deviation (SD) and thin lines representing individual trajectories. Results of two-way mixed-model ANOVA (factors: Time, Group, and Interaction) are indicated in the lower-left corner of each panel. Post-hoc comparisons were performed using Bonferroni’s multiple comparison test: *p < 0.05 vs. pre-game for both groups; # p < 0.05 vs. Game 1 at the same point.

**Supplementary Figure 7 Stronger relationship between HR synchrony and oxytocin in the Low-OT group.**

**A.** Correlation between oxytocin and HR synchrony (CCF) in High-OT group. **B.** Low-OT group. **C**. Correlation between oxytocin and HR synchrony (time lag) in High-OT group. **D.** Low-OT group. Pearson's product-moment correlation coefficient and the results of the single regression analysis are shown in the upper-left corner of each graph. The computed regression line and 95% confidence intervals are shown as bands on the graph. *: p < 0.05.

**Supplementary Figure 8 Graphical model of how sports spectating may foster enjoyment-based social connection via socio-physiological responses.**

Sports spectating is associated with baseline-dependent oxytocin dynamics: individuals with high baseline oxytocin maintain elevated concentrations, whereas those with low baseline levels exhibit increases during spectating. These oxytocin changes were associated with HR synchrony regardless of baseline levels, and the two processes may reciprocally reinforce each other. Such socio-physiological bases ultimately were statistically linked to enjoyment-based social connection among spectators.
